# Quality of oxytocin and tranexamic acid for the prevention and treatment of postpartum hemorrhage in Kenya, Nigeria, South Africa, and Tanzania

**DOI:** 10.1002/ijgo.14197

**Published:** 2022-06-28

**Authors:** Anne Ammerdorffer, Sara Rushwan, Rebecca Timms, Philip Wright, Leanne Beeson, Adam J. Devall, Kristie‐Marie Mammoliti, Fadhlun M. Alwy Al‐beity, Hadiza Galadanci, G. Justus Hofmeyr, Mandisa Singata‐Madliki, Zahida Qureshi, Pete Lambert, Ioannis D. Gallos, Arri Coomarasamy, A. Metin Gülmezoglu

**Affiliations:** ^1^ Concept Foundation Geneva Switzerland; ^2^ Institute of Metabolism and Systems Research (IMSR) WHO Collaborating Centre for Global Women’s Health University of Birmingham Birmingham UK; ^3^ Drug Delivery Disposition and Dynamics, Monash Institute of Pharmaceutical Sciences Monash University Parkville Victoria Australia; ^4^ Department of Obstetrics and Gynecology Muhimbili University of Health and Allied Sciences Dar es Salaam Tanzania; ^5^ Africa Center of Excellence for Population Health and Policy Bayero University Kano Kano Nigeria; ^6^ Department of Obstetrics and Gynecology University of Botswana Gaborone Botswana; ^7^ University of the Witwatersrand Johannesburg South Africa; ^8^ Walter Sisulu University South Africa; ^9^ Effective Care Research Unit, Department of Obstetrics and Gynaecology Universities of the Witwatersrand East London South Africa; ^10^ Department of Obstetrics and Gynecology, School of Medicine, University of Nairobi Kenyatta National Hospital Campus Nairobi Kenya

**Keywords:** Kenya, Nigeria, oxytocin, postpartum hemorrhage, quality assessment, South Africa, Tanzania, tranexamic acid

## Abstract

**Objective:**

To check the quality of oxytocin and tranexamic acid—two recommended products for prevention and treatment of postpartum hemorrhage (PPH)—used in facilities taking part in an implementation research project to improve PPH diagnosis and management.

**Methods:**

Between September 2020 and August 2021, oxytocin and tranexamic acid products used in the study facilities in Kenya, Nigeria, South Africa, and Tanzania were collected and transported in cold storage for analysis. Samples were analyzed according to the International (oxytocin) and British Pharmacopeia (tranexamic acid) standards.

**Results:**

Of the 17 unique oxytocin products, 33 individual measurements were made. Only six unique products had adequate content and no related substances exceeding the recommended limits. Of 14 tranexamic acid samples, 10 showed adequate content. One product in Kenya and two products in Nigeria from different manufacturers had a high content of related substances, which classified them as substandard.

**Conclusion:**

While we were unable to investigate the origin regarding poor manufacturing or poor storage or both, the high number of substandard oxytocin samples is of great concern. Most of the tranexamic acid samples had adequate content but the presence of impurities in multiple products is worrying and requires further study.

## Synopsis

A significant number of oxytocin samples are substandard. Tranexamic acid quality is generally adequate across different products.

## 1 INTRODUCTION

Postpartum hemorrhage (PPH) is the leading cause of maternal mortality in low‐income countries and the primary cause of almost 20% of all maternal deaths globally.^1^ PPH is defined as blood loss of 500 ml or more within the first 24 h after birth, and severe PPH as blood loss of 1000 ml or more. Overall, PPH affects about 5% of all women giving birth around the world.^2^

Use of uterotonics, such as oxytocin, (heat stable) carbetocin (prevention only), misoprostol, and ergometrine, plays a central role in the prevention and treatment of PPH. The World Health Organization’s (WHO) main recommendation to prevent PPH is to use oxytocin (10 IU, intramuscularly/intravenously) as it is effective, has minimal adverse effects, no major contraindications, and is inexpensive compared with the other available options.^2^ In 2017, early use of intravenous tranexamic acid (TXA) was recommended for first‐line PPH treatment in addition to the standard care.^3^ TXA is a competitive inhibitor of plasminogen activation; it can reduce bleeding by inhibiting the enzymatic breakdown of fibrinogen and fibrin clots and it has been shown to reduce mortality due to trauma‐related hemorrhage.^4^ Prior to the addition of TXA in the WHO guidelines for PPH treatment, the largest trial of TXA for the treatment of PPH was conducted in almost 200 hospitals in 21 countries with different income levels. The World Maternal Antifibrinolytic (WOMAN) trial included over 20 000 women with a clinical diagnosis of PPH, and the results indicated that intravenous TXA usage within 3 h of birth reduces maternal death due to bleeding.^5^, ^6^ Both oxytocin and TXA are listed on the WHO Essential Medicines List for management of PPH.^7^

In 2016, Torloni et al.^8^ reviewed the quality of oxytocin available in low‐ and middle‐income countries (LMICs). From the 559 samples assessed (8 studies, 15 different countries), over one‐third of the samples had low (<90%) oxytocin content, indicating substandard drugs. In addition, two samples had no active ingredient, which suggests possible counterfeit drugs.^8^ More recent studies revealed the low quality of oxytocin products in the Democratic Republic of the Congo (15 samples, 80% substandard),^9^ Nigeria (159 samples, 74.2% failed),^10^ and Nepal/Vietnam (42 samples, 31.0% failed).^11^ In contrast, there are also studies showing moderate and good quality oxytocin products.^12^, ^13^, ^14^ For example, a study in Malawi and Ethiopia showed 89% and 96% of the samples, respectively, meeting the criteria.^13^, ^14^ A more recent systematic review by Torloni et al.^15^ found that, overall, around 40% of oxytocin samples were substandard. It is recommended that oxytocin products are stored at 2°C–8°C in order to ensure quality.^16^ Reasons for the low oxytocin content could be substandard manufacturing, inadequate distribution and storage conditions, or a combination of these factors. Even though some manufacturers register oxytocin products for storage at 15°C–25°C, recent research indicated that those products have similar degradation profiles as others that are labeled for cold storage.^17^

For TXA, although both innovator and generic products for oral and parenteral use have been on the market for many years, the treatment indication for PPH is relatively recent. To our knowledge, there are currently no published quality assessment studies of TXA.

In 2017, WHO led the development of a PPH first response treatment bundle based on existing recommendations.^18^ The four elements in the bundle include oxytocin, intravenous fluids, TXA, and uterine massage.^18^ Early diagnosis of PPH and the effectiveness of the bundle is the subject of the E‐MOTIVE implementation research.^19^ The E‐MOTIVE project aims to improve the detection and first response management of PPH through the implementation of the “E‐MOTIVE” bundle, which consists of: (1) Early PPH detection using a calibrated drape; (2) uterine Massage; (3) Oxytocic drugs; (4) Tranexamic acid; (5) Intra Venous fluids; and (6) genital tract Examination and escalation when necessary. The E‐MOTIVE project takes place in Kenya, Nigeria, Pakistan, South Africa, and Tanzania.^19^ The aim of the present study was to assess the quality of the oxytocin and TXA products used in the E‐MOTIVE selected health facilities in Kenya, Nigeria, South Africa, and Tanzania.

## 2 MATERIALS AND METHODS

### 2.1 Sample collection

Samples were collected from health facilities in Kenya, Nigeria, South Africa, and Tanzania that participated in the E‐MOTIVE study.^19^ Local ethical approvals for the study, including the shipment and quality measurement of the oxytocin and TXA samples were acquired. Due to budget limitations, we were not able to test oxytocin and TXA from each E‐MOTIVE facility. We were able to test all unique (i.e. distinct products) and additional products from sites that were geographically close to the sample shipment point, especially in Nigeria and Tanzania. The facilities provided 10 ampoules of oxytocin (1 package) and, if available, 10 ampoules of TXA (2 packages). Ampoules came from the same batch, in their original packaging, not opened, and at least 6 months before the expiry date. After local country collection, the samples were shipped to Monash University in Australia at 2°C–8°C in the presence of a temperature logger.

### 2.2 Sample preparation

From each received sample (containing 10 ampoules each for oxytocin and TXA), three oxytocin ampoules and four TXA samples (2 from each package) were prepared for the initial analysis. If the ampoules showed inconsistent results (active ingredient and/or related substances), three additional ampoules were measured. Quantification of oxytocin in solution for injection was conducted as described in the WHO Oxytocin for Injection Monograph (2015–01).^20^ Oxytocin samples were transferred from the ampoule directly to clear‐glass high‐performance liquid chromatography (HPLC) vials wrapped in aluminum foil for HPLC analysis.

Quantification of TXA in solution for injection was conducted as described in the Tranexamic Acid Ph. Eur. Monograph 0875.^21^ TXA samples were diluted 1 in 20 with ultrapure water to bring the concentration of the solution to within the range of the validated HPLC potency assay (2.5–6 mg/ml). The dilutions were performed directly in clear glass HPLC vials wrapped in aluminum foil prior to HPLC analysis.

### 2.3 Assay

All oxytocin and TXA samples were analyzed on a Nexera X2 UHPLC (Shimadzu, Tokyo, Japan). The HPLC assay for analysis of oxytocin is described in the Oxytocin Injection Monograph (2015–01).^20^ The column utilized was a Luna 5 μM C18(2) 100A, LC column, 250 x 4.6 mm (Phenomenex). The validated calibration curve range was 6.68–20.04 μg/ml (40%–120% relative to the nominal label claim of 16.7 μg/ml oxytocin, i.e. 10 IU/ml). The retention time of oxytocin was 22.4 min. The HPLC assay for potency of TXA is based on the TXA‐related substances assay found in Ph. Eur. Monograph 0875.^21^ The column utilized was a ZORBAX Eclipse Plus C18, 4.6 × 250 mm, 5‐micron particle size (Agilent Technologies). The linearity concentration range in the method was extended and validated to 2.5–6 mg/ml for potency determination (50%–120% relative to the nominal label claim of 100 mg/ml, after a 1 in 20 dilution). The retention time of TXA was 11.8 min. Details of the reference standards used for oxytocin and TXA can be found in Table 1.

**TABLE 1:** Reference standard details for oxytocin and tranexamic acid

Reference standard	Name of supplier	Product code	Potency	Batch code
Oxytocin Chemical Reference Standard	European Pharmacopeia	O0700000	250 μg/ml (reconstituted), diluted to 16.7 μg/ml	6
Tranexamic Acid; Pharmaceutical Secondary Standard; Certified Reference Material	Sigma‐Aldrich	PHR1812	99.9%	LRAA8626
Tranexamic Acid Impurity Standard; Chemical Reference Substance	British Pharmacopeia	734	Not declared, not quantitative	3977

### 2.4 Acceptance criteria

The oxytocin acceptance criterion specifies that the product contains no less than 90.0% and no more than 110.0% of the amount of active ingredient stated on the label.^20^ The TXA acceptance criterion specifies that the product contains no less than 95.0% and no more than 105.0% of the amount of active ingredient stated on the label.^21^ The related substance acceptance criteria for oxytocin are no more than one individual impurity greater than 2.0% and total impurities no greater than 5.0%.^20^ The related substance acceptance criteria for TXA are related compound (Cpd) A no more than 1.0%; related Cpd B no more than 0.5%; all other impurities no more than 0.1%. ^21^

## 3 RESULTS

### 3.1 Oxytocin

#### 3.1.1 Number or products

**FIGURE 1:**
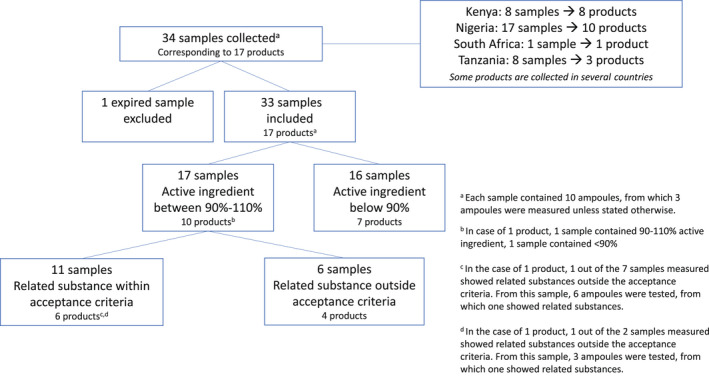
Selection and analysis results of oxytocin samples

Between September 2020 and August 2021, 34 individual oxytocin samples were collected from health facilities in Kenya (8 samples, 8 products), Nigeria (17 samples, 10 products), Tanzania (8 samples, 3 products), and South Africa (1 sample, 1 product) (Figure 1 and Table 2). In Kenya and South Africa, all collected products met the acceptance criteria for the active ingredient, compared with only one‐third of the products used in Nigeria and Tanzania. Related substances were measured in 8 out of 10 products in Nigeria, 3 out of 8 in Kenya, 1 out of 3 in Tanzania, and not in the South African product.

**TABLE 2:** Oxytocin products per country of sample collection

Country of collection	Number of products (# of samples)	Number of products with active ingredient between 90%–110% (% of products)	Number of products with related substances above limit (% of products)
Kenya	8 (8)	8 (100)	3 (38)
Nigeria	10 (17)	4 (40)	8 (80)
Tanzania	3 (8)^a^	1 (33)	1 (33)
South Africa	1 (1)	1 (100)	0 (0)

^a^ One product expired during measurement.

Overall, 17 unique products were identified (Table 3). Oxytocin 3 was collected at seven different facilities: one in Kenya, two in Nigeria, three in Tanzania, and one in South Africa. From these seven samples, two had the same batch number. Two samples were collected from product oxytocin 4 (Kenya and Tanzania), oxytocin 6 (Kenya and Nigeria), oxytocin 10 (two Nigerian facilities, same batch number), and oxytocin 13 (two Nigerian facilities, different batch numbers). Three samples were collected from oxytocin 9 at three facilities in Nigeria (different batch numbers) and oxytocin 12 (three Nigerian facilities, different batch numbers). Oxytocin 17 was collected from four Tanzanian facilities, consisting of two different batch numbers.

**TABLE 3:** Details of oxytocin samples

Unique product	Sample number^a^	Country of collection	Country of manufacturer	WHO prequalified or approved by SRA	Recommended storage condition	Reported storage condition	Primary ampoule description	Date of manufacture	Expiry date	Batch number	Date measured	Strength	Active ingredient complies 90%–110%^b^	Related substances^c^
1	OXY1	Kenya	India	No	2–8°C	4°C	Clear glass	2/2020	1/2022	ML20080	6/2021	10 IU/1 ml	Yes (92.8–98.6)	Yes, 3 peaks (1/3)
												Additional measurement 3 ampoules	Yes (94.5–98.7)	Yes, 1 peak (1/3), 2 peaks (2/3)
2	OXY2	Kenya	India	No	2–8°C	4°C	Clear glass	2/2020	1/2022	0740	6/2021	10 IU/1 ml	Yes (100.7–100.9)	No
3	OXY3.a	Kenya	Germany	SRA	2–8°C	4°C	Clear glass	4/2020	4/2023	00307	6/2021	10 IU/1 ml	Yes (96.3–96.8)	No
	OXY3.b	Nigeria	Germany	SRA	2–8°C	RT, 24°C	Clear glass	4/2019	4/2022	90 479	11/2020	10 IU/1 ml	Yes (92.0–92.4)	No
	OXY3.c	Nigeria	Germany	SRA	2–8°C	Refrigerated	Clear glass	6/2019	6/2022	90 660	11/2020	10 IU/1 ml	Yes (93.1–93.2)	No
	OXY3.d	Tanzania	Germany	SRA	2–8°C	3°C	Clear glass	11/2019	11/2022	90 820	6/2021	10 IU/1 ml	Yes (99.1–99.6)	Yes, 2 peaks (1/3)
												Additional measurement 3 ampoules	Yes (96.9–97.7)	No
	OXY3.e	Tanzania	Germany	SRA	2–8°C	Not reported	Clear glass	6/2019	6/2022	90 660	6/2021	10 IU/1 ml	Yes (99.7–100.3) ^d^	No
	OXY3.f	Tanzania	Germany	SRA	2–8°C	4°C	Clear glass	1/2020	1/2023	90 879	6/2021	10 IU/1 ml	Yes (99.4–99.7)	No
	OXY3.g	South Africa	Germany	SRA	2–8°C	Not reported	Clear glass	1/2020	1/2023	90 880	10/2020	10 IU/1 ml	Yes (98.8–98.4)	No
4	OXY4.a	Kenya	India	No	8–25°C	4°C	Clear glass	9/2019	8/2021	V19226	6/2021	10 IU/1 ml	Yes (91.6–92.0)	No
	OXY4.b	Tanzania	India	No	8–25°C	Not reported	Clear glass	3/2019	2/2021	V19077	6/2021	5 IU/1 ml	No (86.5–87.1)	Yes, 2–3 peaks (3/3)
5	OXY5	Kenya	India	No	2–8°C	4°C	Amber glass	6/2020	5/2022	0 EB05262	6/2021	10 IU/1 ml	Yes (101.4–101.8)	Yes, 2 peaks (1/3)
												Additional measurement 3 ampoules	Yes (100.4–101.4)	Yes, 2 peaks (3/3)
6	OXY6.a	Kenya	Switzerland	SRA	2–8°C	4°C	Clear glass	5/2018	9/2021	SMR66	6/2021	5 IU/1 ml	Yes (97.3–97.5)	No
	OXY6.b	Nigeria	Switzerland	SRA	2–8°C	Refrigerated	Clear glass	1/2018	12/2022	SMJ61	11/2020	10 IU/1 ml	Yes (94.8–94.8)	No
7	OXY7	Kenya	India	No	2–8°C	4°C	Amber glass	10/2019	9/2021	9EA05277	6/2021	10 IU/1 ml	Yes(94.3–95.5)	No
8	OXY8	Kenya	India	No	2–8°C	3.7°C	Clear glass	8/2020	7/2022	KLOY0008	6/2021	10 IU/1 ml	Yes (99.6–107.9)	Yes, 2 peaks (1/3)
												Additional measurement 3 ampoules	Yes (99.6–100.7)	No
9	OXY9.a	Nigeria	China	No	Cool place	Shelf (not specified)	Clear glass	11/2018	10/2021	181 101 10	11/2020	10 IU/1 ml	No (74.0–74.9)	Yes, >50 peaks (3/3)
	OXY9.b	Nigeria	China	No	Cool place	25°C	Clear glass	11/2018	10/2021	181 101 04	11/2020	10 IU/1 ml	No (70.4–70.7)	Yes, >50 peaks (3/3)
	OXY9.c	Nigeria	China	No	Cool place	Not reported	Clear glass	11/2018	10/2021	181 101 06	11/2020	10 IU/1 ml	No (69.3–73.8)	Yes, >50 peaks (3/3)
10	OXY10.a	Nigeria	Chile	No	<25°C	0–4°C	Clear glass	6/2018	6/2021	75MF0914	11/2020	10 IU/1 ml	No(84.8–84.9)	Yes, 3 peaks (3/3)
	OXY10.b	Nigeria	Chile	No	<25°C	20°C	Clear glass	6/2018	6/2021	75MF0914	11/2020	10 IU/1 ml	No (83.2–83.3)	Yes, 4 peaks (3/3)
11	OXY11	Nigeria	Nigeria	No	No packaging/label	Refrigerated	Clear plastic	8/2018	7/2021	75HH01	11/2020	10 IU/2 ml	No (81.7–81.8)	Yes, 5 peaks (3/3)
12	OXY12.a	Nigeria	China	No	<25°C	Refrigerated −8 to 8°C	Clear glass	3/2019	3/2022	190 315	11/2020	10 IU/1 ml	No (82.2–87.0)	Yes, 3 peaks (3/3)
	OXY12.b	Nigeria	China	No	<25°C	Refrigerated	Clear glass	5/2019	5/2022	190 518	11/2020	10 IU/1 ml	No (81.6–82.4)	No
	OXY12.c	Nigeria	China	No	< 25°C	Refrigerated	Clear glass	11/2019	11/2022	191 108	09/2021	10 I.U./1 ml	No(82.3–82.5)	Yes, 2 peaks (3/3)
13	OXY13.a	Nigeria	China	No	Cool place	Not reported	Clear glass	6/2019	5/2022	190 613	11/2020	10 IU/1 ml	No (86.3–87.1)	Yes, >50 peaks (3/3)
	OXY13.b	Nigeria	China	No	Cool place	Refrigerated	Clear glass	10/2019	09/2022	191 017	09/2021	10 I.U./1 ml	Yes(92.8–93.1)	Yes, >50 peaks (3/3)
14	OXY14	Nigeria	China	No	<25°C	Refrigerated	Clear glass	4/2020	4/2023	200 415	09/2021	10 I.U./1 ml	Yes(104.3–105.3)	Yes, >50 peaks (3/3)
15	OXY15	Nigeria	China	No	<25°C	Refrigerated	Clear glass	5/2020	5/2023	200 569	09/2021	10 I.U./1 ml	No(84.4–84.5)	Yes, 3 peaks (2/3), 2 peaks (1/3)
16	OXY16	Nigeria	India	No	2–8°C	RT (27°C)	Amber glass	9/2020	8/2023	20OA06	09/2021	10 I.U./1 ml	No (85.0–86.0)	Yes, 4 peaks (2/3), 3 peaks (1/3)
17	OXY17.a	Tanzania	India	No	2–8°C	4°C	Clear glass	4/2020	3/2022	5D00127	6/2021	10 IU/1 ml	No (89.0–89.2)	No
	OXY17.b	Tanzania	India	No	2–8°C	6°C	Clear glass	4/2020	3/2022	5D00127	6/2021	10 IU/1 ml	No (88.9–89.6)	No
	OXY17.c	Tanzania	India	No	2–8°C	7°C	Clear glass	4/2020	3/2022	5D00124	6/2021	10 IU/1 ml	No (88.7–89.2)	No
	OXY17.d	Tanzania	India	No	2–8°C	5°C	Clear glass	4/2020	3/2022	5D00124	6/2021	10 IU/1 ml	No (88.0–88.6)	No

Abbreviation: RT, room temperature.

^a^ If multiple samples were collected from one unique oxytocin product, this is indicated by an additional letter behind the sample number.

^b^ The percentage range of three measured ampoules is given in brackets.

^c^ (x/x) Number of ampoules with related substances versus total number of ampoules measured.

^d^ Two ampoules measured.

#### 3.1.2 Product details

From the 17 unique products, eight were manufactured in India, five in China, and one product each in Chile, Germany, Nigeria, and Switzerland. None of the products manufactured in Chile and Nigeria met the acceptance criteria and related substances were observed in all of these products. In two Chinese manufactured products, the acceptance criteria for the active ingredient content were met, though these products showed large amounts of related substances. Most of the products manufactured in India (88%) contained the active ingredient between 90%–110%, although related substances outside the acceptable limits were observed in four out of eight of the products. The two products from Germany and Switzerland met the acceptance criteria and showed no related substances. None of the 17 oxytocin products are prequalified by WHO, but oxytocin 3 and oxytocin 6 were WHO‐Listed Authority (WLA) or Stringent Regulatory Authority (SRA) approved.^22^

The recommended storage conditions were 2°C–8°C (9 products), 8°C–25°C (one product), <25°C (4 products), to be stored at a cool place (temperature not defined, two products), or unknown (one product). Most of the health facilities that self‐reported the storage conditions of their oxytocin products complied with these recommendations. Several samples were not stored under the recommended storage conditions: three samples were kept at room temperature instead of 2°C–8°C or a cool place, and two samples were kept colder than recommended. Five facilities did not report their local storage conditions.

The primary packaging was a clear glass ampoule for most products, while three products were packaged in an amber glass ampoule, and one product was packaged in a clear plastic ampoule.

The quality of three samples was measured while their expiry date was within 6 months. One Tanzanian sample had expired 4 months previously at the time of measurement and was excluded from the analysis. Most of the samples had a strength of 10 IU/1 ml, except for product oxytocin 11, which had 5 IU/1 ml. In the case of product oxytocin 4 and 6, one sample had a strength of 10 IU/1 ml, while the second sample had a strength of 5 IU/1 ml.

#### 3.1.3 Quality assessment: active ingredient and related substances

Of the 17 products, oxytocin content in 10 products was within 90%–110% (Table 3). The active ingredient content varied across the other products, ranging between 69.3% and 89.2%.

Seven products contained related substances above the recommended limit in all samples measured. The number of related substances in oxytocin 9, oxytocin 13, and oxytocin 14 exceeded 50 peaks. In the case of product oxytocin 1, oxytocin 5, and oxytocin 8, from which only one sample was collected, and six ampoules analyzed, related substances exceeding the limit were measured in respectively four, four, and one ampoule(s). Product oxytocin 3, from which seven samples were measured, showed only related substances above the limit in one sample (1 ampoule out of 6 measured).

### 3.2 Tranexamic acid

#### 3.2.1 Number or products

**FIGURE 2:**
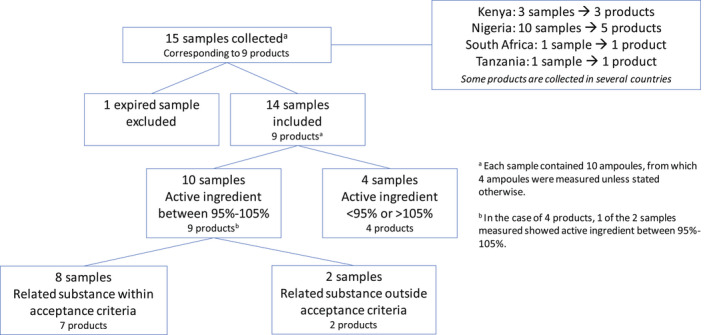
Selection and analysis results of tranexamic acid samples

In total, 15 individual TXA samples were collected from health facilities in Kenya (3 samples), Nigeria (10 samples), Tanzania (1 sample), and South Africa (1 sample) (Figure 2 and Table 4). One Nigerian sample was expired and excluded from analysis, and 9 unique products were identified.

**TABLE 4:** Details of tranexamic acid samples measured

Unique product	Sample number^a^	Country of collection	Country of manufacturer	WHO prequalified or SRA	Recommended storage condition	Reported storage condition	Primary ampoule description	Date of manufacture	Expiry date	Batch number	Date measured	Strength	Active ingredient complies 95–105%^b^	Related substances^c^
1	TXA1.a	Kenya	India	No	<30°C	27°C	Clear glass	10/2019	9/2021	K0AA5004	6/2021	100 mg/ml	Yes (99.5–100.4)	No
	TXA1.b	Tanzania	India	No	<30°C	Not reported	Clear glass	4/2020	3/2022	O2AAT002	6/2021	100 mg/ml	Yes (100.3–101.0)	No
2	TXA2	Kenya	India	No	<30°C	RT/21°C	Clear glass	4/2019	3/2022	ELF8AW0003	6/2021	500 mg/5 ml	Yes (98.1–98.7)	No
3	TXA3	Kenya	Pakistan	No	<30°C	Good/NA	Clear glass	11/2019	10/2022	085	6/2021	500 mg/5 ml	Yes(100.9–102.1)	Yes, 3 peaks (4/4)
4	TXA4.a	Nigeria	India	No	<30°C	RT	Clear glass	8/2019	7/2022	PX‐1908	11/2020	500 mg/5 ml	No	No
	TXA4.b	Nigeria	India	No	<30°C	Not reported	Clear glass	2/2019	1/2022	PX‐1901	11/2020	500 mg/5 ml	Yes (102.2–102.9)	No
5	TXA5	Nigeria	India	No	Cool place	Cold room, 19°C	Clear glass	7/2019	6/2022	AAE‐9044	11/2020	100 mg/ml	Yes (101.5–103.1)	No
6	TXA6.a	Nigeria	India	No	<25°C	Not reported	Clear glass	2/2019	1/2022	N‐13285	11/2020	100 mg/1 ml	Yes (102.1–102.7)	No
	TXA6.b	Nigeria	India	No	<25°C	20°C	Clear glass	8/2019	7/2022	N‐14424	11/2020	100 mg/ml	No	No
7	TXA7.a	Nigeria	India	No	Cool place	RT	Amber glass	5/2018	4/2021	1 006 001	11/2020	500 mg/5 ml	No	Yes, 1 peak (4/4)
	TXA7.b	Nigeria	India	No	Cool, dry, and dark place	RT	Amber glass	01/2021	12/2023	1 273 001	9/2021	500 mg/5 ml	Yes (95.4–96.5)	Yes, 1 peak (4/4)
8	TXA8.a	Nigeria	India	No	Cool, dry, and dark place	RT	Amber glass	12/2020	11/2023	SA1‐10719	9/2021	100 mg/ml	No	Yes, 1 peak (4/4)
	TXA8.b	Nigeria	India	No	Cool, dry, and dark place	RT	Amber glass	7/2020	6/2022	SA1‐9476	9/2021	100 mg/ml	Yes (98.6–100.0)	No
9	TXA9	South Africa	South Africa	SRA	<25°C	Not reported	Clear glass	2/2019	1/2022	AW1779	10/2020	500 mg/5 ml	Yes(100.2–101.5)	No

Abbreviation: RT, room temperature.

^a^ If multiple samples were collected from one unique TXA product, this is indicated by an additional letter behind the sample number.

^b^ The percentage range of four measured ampoules is given in brackets.

^c^ (x/x) Number of ampoules with related substances versus total number of ampoules measured.

^d^ Two ampoules measured.

#### 3.2.2 Product details

Most of the products were manufactured in India (7 out of 9), with the remaining two in Pakistan and South Africa. The recommended storage conditions were <25°C, <30°C, or to store in a cool place (temperature not defined). Health facilities self‐reported that they complied with the storage conditions. Most of the products were in clear glass ampoules, while two products were in an amber glass ampoule. None of the products were expired at the time of quality measurement, although the expiry date of two samples was within 6 months. The strength of all products was 100 mg/ml, and the volume of the ampoule 5 ml.

#### 3.2.3 Quality assessment: active ingredient and related substances

The concentration of TXA in 10 of 14 samples analyzed was within ±5% of the specified label claim and met the acceptance criterion (Table 4). The concentration of two of the samples was found to be outside the lower end of the specification by less than 1%, and the concentration of the remaining two samples exceeded the upper end of the specification by less than 2%. Comparing the presence of related substances of the 14 samples versus the related substances acceptance criteria, none of the samples contained unacceptable levels of Cpd A and Cpd B. However, three samples contained large amounts of unknown substances. Product TXA3 (all four ampoules) contained large unknown impurity peaks at retention times of 5.7, 7.3, and 26.2 min, and a smaller impurity peak at 8.0 min. Product TXA7 (all eight ampoules) contained a large unknown impurity peak at a retention time of 5.7–6.2 min. Product TXA8 (sample TXA8.a, four ampoules) contained a large unknown impurity peak at a retention time of 5.7 min.

## 4 DISCUSSION

The present study provides further evidence of widespread presence of substandard oxytocin products. This is the first published analysis of TXA, and it is noteworthy that the active ingredient content was within required limits in all products; however, the presence of related substances is of concern.

Most of the oxytocin products collected in Nigeria (80%) did not meet the quality criteria. A similar percentage was observed by Anyakora et al.,^10^ where 74.2% of the 159 samples failed the criteria. In our study, SRA approved products collected in Nigeria met the criteria, while in the Anyakora et al.^10^ study, SRA‐approved product samples also showed low active oxytocin content. Only one of the non‐SRA approved products in Nigeria contained the specified content of active ingredient (oxytocin product 14) and almost all products had related substances above the accepted limits. In Kenya, where the collected products came from India, Germany, or Switzerland, none failed the criteria; however, one‐third of the samples showed related substances exceeding the accepted limits. Substandard oxytocin product quality in Kenya and Tanzania has also been reported.^23^

Both innovator and generic TXA products for oral and parenteral use have been on the market for many years; however, the treatment indication for PPH is relatively recent. While there are many TXA products available, only one collected in this study was WLA–SRA approved. The current study is the first to report on the quality of TXA products used for PPH treatment in Kenya, Nigeria, South Africa, and Tanzania. Overall, 10 of 14 TXA samples analyzed complied with the acceptance criteria for active ingredient content (95%‐105% of nominal content). While the concentration of the remaining four samples showed minor deviations from this range, none of the nine TXA products tested showed any substantive issues related to active ingredient content. No counterfeit drugs were detected. A major advantage of TXA is that no cold chain is needed to ensure the quality of the product. Products were kept between approximately 19°C and 27°C at the facilities.

In three products, large peaks of unknown related substances were observed (Table 4). While the details of these peaks have not been further analyzed, these three products were considered substandard. Since the TXA molecule is considered stable and that quality issues related to TXA samples were not expected, further investigation on TXA quality is required. During collection of oxytocin and TXA samples, it became clear that TXA availability is lower in the facilities and TXA more often needs to be obtained privately by the patient’s relatives.

Like most other studies that examined the quality of oxytocin, the products were collected at the end of the supply chain, namely either at the pharmacy or more often at the labor ward of the health facility. This is a limitation of the study, as the quality of the samples has not been measured directly upon manufacturing, during distribution, and at the local storage environment (prelabor ward). Even though most of the health facilities self‐reported cold storage of their oxytocin, it is reasonable to question whether consistent 2°C–8°C storage had been guaranteed, as knowledge of cold oxytocin storage is not always prevalent among healthcare providers, policy makers, and supply chain experts.^24^ We were not able to verify the reported storage conditions by visiting the facilities and observing the actual locations due to COVID‐19‐related challenges. Furthermore, with 17 products (of which 10 included only 1 sample measurement), the number of oxytocin products was limited and does not represent the overall national situation in each country. However, the results of the study do indicate that there are many low‐quality oxytocin products in use, which needs to be addressed to realize the desired decrease in PPH morbidity and mortality. Finally, this study was a funded implementation research study and met great challenges in sample collection and transport within countries and internationally. COVID‐19‐pandemic‐related travel challenges further complicated sample collection and transport. We used temperature logs with each transport and established no serious temperature excursions. It is worth noting that these quality assessments are labor intensive, time consuming, and expensive.

### 4.1 Clinical implications

Quality issues surrounding generic medicines used in low‐ and middle‐income countries are not new. However, substantial quality issues remain. From a clinical perspective, all oxytocin should be kept in cold chain and storage (2°C–8°C) and providers should be aware if the brand is quality‐assured, i.e. WHO prequalified (https://extranet.who.int/pqweb/medicines/prequalified‐lists/finished‐pharmaceutical‐products) or approved by a stringent regulatory authority for reassurance. There is high probability of being substandard if quality assured manufacture is not confirmed. The use of multiple doses of oxytocin or oxytocin and misoprostol are known but their safety and efficacy are unknown. Although not tested in the present study, significant quality issues are also common with misoprostol. We do not think that combinations of uterotonics or doubling or tripling of oxytocin are effective ways of overcoming quality concerns.

In conclusion, we strongly recommend the use of quality‐assured uterotonics and, in parallel, development of low‐cost quality assessment technologies so that substandard products can be more readily recognized. Our findings should provide a further motivation for policymakers to ensure that WHO‐Listed Authority/SRA approved products are always purchased, and that oxytocin is always kept in cold chain and storage.

## CONFLICT OF INTEREST

Related to the present study AA, AMG, and SR report a BMGF grant to the University of Birmingham for Concept Foundation. Outside the study, AA, AMG, and SR report a grant from the BMGF to Concept Foundation for landscaping the need for accelerating innovation for mothers. AA, AMG, and SR report funding from MSD for Mothers paid to the Concept Foundation. AMG reports an honorarium from Ferring Pharmaceuticals paid to Concept Foundation for PPH panel participation (International Confederation of Midwives Conference, June 23, 2021). PL reports grant funding from Johnson & Johnson and the Victoria Medical Research Acceleration Fund to Monash University to support development of a heat‐stable inhaled oxytocin product for management of PPH in low‐resource settings. Other authors report no conflicts of interest.

## AUTHOR CONTRIBUTIONS

IDG and AC conceptualized the study. IDG, AC, and AMG initiated the project. AA, SR, RT, PW, LB, AJD, KMM, FMAA, HG, GJH, ZQ, PL, IDG, AC, and AMG contributed to the acquisition, analysis, and interpretation of the findings. AA wrote the draft manuscript. All authors approved the final version.
